# Pharmacophore and docking-based sequential virtual screening for the identification of novel Sigma 1 receptor ligands

**DOI:** 10.6026/97320630015586

**Published:** 2019-09-10

**Authors:** Mubarak A Alamri, Mohammed A Alamri

**Affiliations:** 1 Department of Pharmaceutical Chemistry, College of Pharmacy, Prince Sattam Bin Abdulaziz University, Alkharj 11942, Saudi Arabia; 2 Department of Pharmacology, College of Pharmacy, Prince Sattam Bin Abdulaziz University, Alkharj 11942, Saudi Arabia

**Keywords:** Sigma 1, Sigma 2, pharmacophore modelling, screening, molecular docking

## Abstract

Sigma 1 receptor (σ1), a small transmembrane protein expressed in most human cells participates in modulating the function of other membrane proteins such as G protein coupled receptors and ion channels. Several ligands targeting this receptor are currently in clinical trials for the treatment of Alzheimer's disease, ischemic stroke and neuro-pathic pain. Hence, this receptor has emerged as an attractive target for the treatment of neuro-pathological diseases with unmet medical needs. It is of interest to identify and characterise novelσ1 receptor ligands with different chemical scaffolds using computer-aided drug designing approach. In this work, a GPCR-focused chemical library consisting of 8543 compounds was screened by pharmacophore and docking-based virtual screening methods using LigandScout 4.3 and Autodock Vina 1.1.2 in PyRx 0.8, respectively. The pharmacophore model was constructed based on the interactions of a selective agonist and another antagonist ligand with high binding affinity to the human σ1receptors. Candidate compounds were filtered sequentially by pharmacophore-fit scores, docking energy scores, drug-likeness filters and ADMET properties. The binding mode and pharmacophore mapping of candidate compounds were analysed by Autodock Vina 1.1.2 and LigandScout 4.3 programs, respectively. A pharmacophore model composed of three hydrophobic and positive ionizable features with recognized geometry was built and used as a 3D query for screening a GPCR-focused chemical library by LigandScout 4.3 program. Among the screened 8543 compounds, 159 candidate compounds were obtained from pharmacophore-based screening. 45 compounds among them bound to σ 1receptor with high binding-affinity scores in comparison to the co-crystallized ligand. Amongst these, top five candidate compounds with excellent druglikeness and ADMET properties were selected. These five candidate compounds may act as potential σ1 receptor ligands.

## Background

The discovery of sigma receptors was initiated in 1976 by Martin, et al. Experimentation with synthetic opioids eluted to the existence of new and unknown receptor(s) causing unpredictable psycho activity [1]. Early characterization of the different receptor subtypes (1 and 2) was based on ligand selectivity and protein mass [2-5]. The sigma-1 (σ1) receptor was sequenced, cloned, and had shown unique pharmacological attributes in multiple studies [6-13]. Recent studies revealed the crystal structure and topology of the σ1 receptor, which was shown to have transmembrane domains with a single-pass structure [14]. The sub cellular localization of the σ1 receptor shows residence in lipid raft-like domains in endoplasmic reticulum where it is thought to act as a chaperone protein [15,16]. Physiologically, σ1 receptors are heavily involved with ion channels where they are found to interact with inositol trisphosphate receptors (Ca+2 channels), multiple voltage-gated K+ channels, as well as volume-regulated Cl- channels [17-22]. The involvement of σ1 receptors with multiple secondary messengers is further reflected in its ability to influence the signalling of several neurotransmitters such as serotonin, dopamine, and glutamate as well as neuronal growth factors [23-25].Furthermore, σ1 receptors were found to be involved in several inflammatory pathways [26-28]. And the internalization of σ1 receptors into intracellular compartments was shown to be in an active manner [29]. This wide range of activity made σ1 receptors involved and targeted in many disorders such as cancer and retinal neural degeneration, in addition to a host of abnormal CNS conditions such as Alzheimer's, schizophrenia, depression, and addiction [30-39]. There is currently no known endogenous ligand exclusive to the σ1 receptor, but multiple molecules have shown interaction with the σ1 receptor with varying affinities and functionalities (i.e. agonistic or antagonistic activity). Examples of these endogenous ligands are dimethyl tryptamine, sphignosines, dehydro epiandrosterone, pregnenolone, and progesterone [40-43]. Many compounds with high affinity for σ1 receptors such as haloperidol (antagonist), fluvoxamine, (+)-pentazocine, and dextromethorphan have been used as valuable research tools, as well as antipsychotics, antidepressants, neuroprotectants, and anti tussives [44-48]. Currently, σ1 receptor agonists and antagonists are in clinical trials for treatment of Alzheimer’s disease, ischemic stroke, and neuropathic pain [49-51]. Thus, identification of new compounds targeting theσ1 receptor may yield selective ligands that will further enable us to understand and treat conditions where σ1 receptors underlie the disease.

In modern drug discovery, virtual screening of chemical databases is a significant tool to identify new lead compounds to modulate the activity of a particular target in time and cost-effective manners. This computer-aided drug designing approach is broadly classified into ligand-based and structure-based drug designing which depends on the information available about the ligands and protein structure (3D), respectively [52]. Recently, the structures of σ1 receptor in complex with different ligands have been reported, which facilitated the application of structure-based approach for identifying ideal pharmacophore against σ1 receptor[14].Therefore, in this study, we approached the search for potential novel and chemically diverse σ1 receptor ligands by pharmacophore and docking-based sequential virtual screening of a GPCR-focused chemical library against σ1 receptor using ligandScout 4.3 and AutoDock Vina 1.1.2 programs [53,54].

## Materials and Methods:

**Figure 1:**
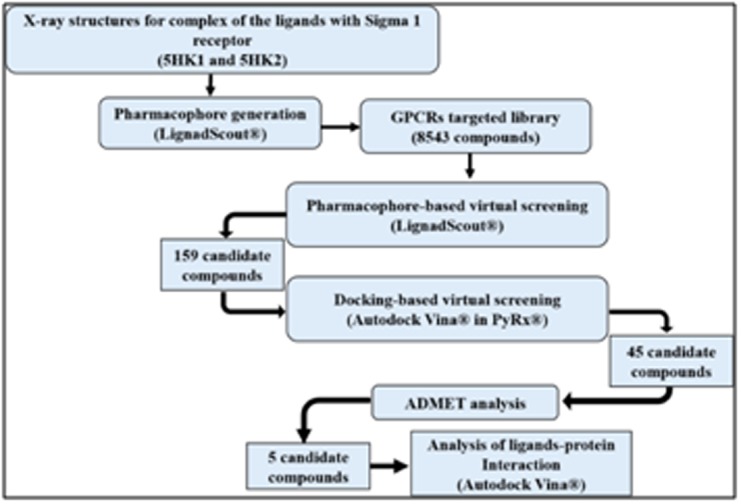
Schematic representation of the computational workflow for this study.

The methodology used in this research is shown in Figure 1.

## Generation of pharmacophore model:

Pharmacophore describes the spatial arrangement of essential interactions in a receptor-binding site. Structure-based pharmacophore method deals with the three-dimensional structure of macromolecules-ligand complex. The key chemical features of the ligand binding pocket along with their spatial relationship are considered to generate a pharmacophore model [55]. In this context, LigandScout4.3 program was used to construct a pharmacophore model based on two X-ray crystal structures of human σ1 receptors in complex with two different ligands. The PDB entries for these structures are 5HK1 and 5HK2 with an X-ray resolution of 2.5051 and 3.2 Å, respectively. X-ray structures were obtained from protein data bank [56].5HK1 and 5KH2 are 3D structures of σ1receptor in complex with PD144418 and 4-IBP ligands, respectively [14]. Initially, two separate pharmacophores were generated from the interaction of these ligands with σ1 receptor using pharmacophore generation tool in LigandScout4.3 and then both pharmacophore hypotheses were aligned to extract the shared features. Finally, the exclusion volume was added to the final pharmacophore model to be used as 3D query features for virtual screening of chemical database.

## Selection of chemical library:

In silico virtual screening was performed with "Life Chemicals GPCR Targeted Library" having 8543 ligand molecules to identify lead molecules with high binding affinity toward σ1 receptor. The chemical library was retrieved from Life Chemicals [57].

## Pharmacophore-based virtual screening:

LigandScout4.3 program was used to carry out the structure-based pharmacophore virtual screening of 8543 ligand molecules against the corresponding pharmacophore model using a default setting. LigandScout4.3 was used initially to convert the ligands SDF files into PDB. The obtained hits from screening exercises were ranked based on their pharmacophore fit scores.

## Docking-based virtual screening:

Autodock vina 1.1.2 in PyRx 0.8 was used to perform the dockingbased virtual screening of 159 candidate compounds against the Xray structure of human σ1 receptor (PDB: 5HK1) [58].Initially, PyRx was used for energy minimization of compounds and for converting all molecules to AutoDock Ligand format (PDBQT). Compounds with binding energy score better than the original ligand (PD144418), 45 compounds, were considered for further investigation.

## Drug-likeness and ADMET properties:

The obtained candidate compounds from sequential virtual screening were subjected to ADMET (absorption, distribution, metabolism, elimination and toxicity) analysis. Swiss ADME web server was used to calculate the drug-likeness parameters according to Lipinski's rule of five as well as the ADME properties [59]. The ProTox-II-Prediction of Toxicity of Chemicals web server was used to predict the hepatotoxicity and mutagenicity of compounds [60]. Compounds only with zero violation of drug-like filters and having good blood brain barrier penetration and aqueous solubility as well as unlikely to cause mutagenicity and dose-dependent hepatotoxicity were picked out as final candidate compounds.

## Molecular docking method:

In order to understand how these ligands bind to σ1 receptor, the five candidate compounds were considered for docking studies. Autodock Vina 1.1.2 was used to perform molecular docking of potential hits against X-ray structure of human σ1 receptor (PDB: 5HK1). Initially, the protein structure was prepared for docking by removing unwanted water molecules and bound ligands from protein structure and adding the polar hydrogen atoms using Discovery Studio Visualizer 2019 [61]. In addition, the same program was used to prepare the co-crystalized ligand and to convert the files into PDB formats. The three-dimensional Grid box for molecular docking simulation was obtained using Autodock tools 1.5.6 [62]. The Grid box was centred to cover the active binding site and all essential residues. Autodock tools program was also used to convert protein and ligand files into PDBQT formats. To validate the parameters of the docking approach, the crystallized ligand, PD144418, was re-docked into the binding pocket and the resulted pose was overlaid over the crystallized one to predict the binding mode.

## Results and Discussion

**Figure 2:**
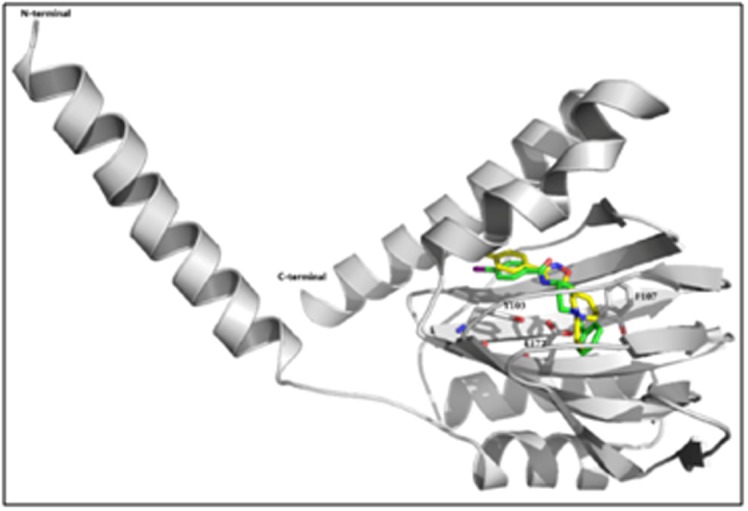
Ribbon representation of sigma 1 receptor structure showed the binding modes of PD144418 (yellow) and 4-IBP (green).

The final structure-based pharmacophore was generated from the shared features of two pharmacophore models constructed from Xray structures of two ligands, antagonist and agonist, bound to the active site of human σ1 receptors. This pharmacophore modelling approach was utilized as it was difficult to distinguish between antagonist and agonist ligands of σ1 receptors based on the binding data without obtaining the functional data. Both types of ligands bind to the same binding site, adapt similar binding mode and interact with almost the same residues in Figure 2 [63]. Therefore, generating the model based on the complex of σ1receptor with ligands having paradoxical actions could increase the efficiency of pharmacophore design.

**Figure 3:**
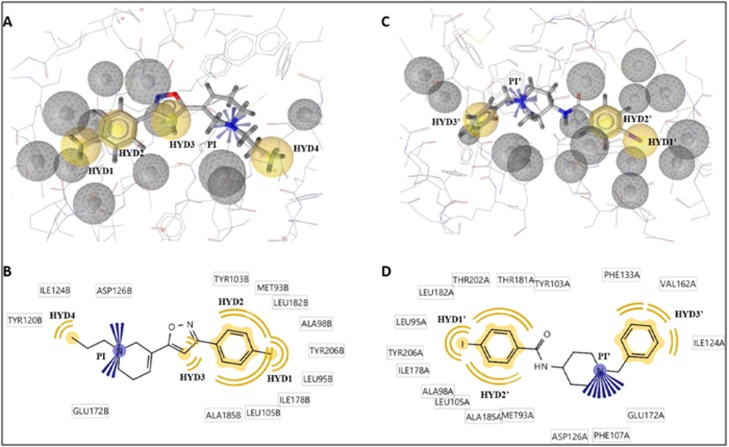
Pharmacophore models derived from two X-ray structures of human σ1 receptors in complex with PD144418 and 4-IBP (PDB: 5HK1 and 5HK2), respectively. (A) and (C) illustrates pharmacophore models generated with LigandScout software from PDB 5HK1 and 5HK2, respectively. The pharmacophore features were represented in LigandScout by color codes in which, hydrophobic, ionizable positive charge and exclusion volume are depicted as yellow spheres, blue stars and gray spheres, respectively. (B and D) illustrated the 2D interactions of PD144418 and 4-IBP with the binding site residues of σ1 receptor, respectively. HYD and PI stand for hydrophobic and positive ionizable features, respectively.

The first pharmacophore was based on the interaction of PD144418, a high affinity and selective σ1 antagonist, bound to the active site of σ1 receptor (PDB: 5HK1) with a Ki value of 0.08 nM [14]. This pharmacophore comprised of four hydrophobic regions and a positive ionizable group besides twelve exclusion volume spheres that depicted the restricted regions defining the overall shape of the binding pocket (Figure 3A). The hydrophobic features (HYD1, HYD2 and HYD3) are located in a hydrophobic pocket composed by Tyr103, Met93, Leu182, Ala98 Tyr206, Leu95, Ile178, Leu105 and Ala185 which is occupied by the toluene group and the isoxazol ring (Figure 3B). The fourth hydrophobic feature (HY4) is located in a hydrophobic pocket constituted by Tyr120, Ile124 occupied by the methyl group within the propyl side chain. The positive ionizable tertiary amine group (PI) is located in close proximity to Glu172 and Asp126. In the same manner, the second pharmacophore was generated from the structure of 4-IBP, a high affinity and selective σ1 agonist, bound to the same active site of σ1 receptor (PDB:5HK2) with a Ki value of 1.7 nM [14]. The latter pharmacophore was made up of three hydrophobic regions, a positive ionizable group and sixteen exclusion volume spheres (Figure 3C). The hydrophobic features (HYD1', HYD2') are located in a hydrophobic pocket formed by Tye103, Thr181, Thr202, Leu182, Leu95, Tyr206, Ile178, Ala98, Leu105, Ala185 and Met93which is occupied by the iodobenzene group. The third hydrophobic feature (HYD3') is located in another hydrophobic pocket composed by Phe133, Val162 and Ile124 that adapted the benzene ring. Similarly, the positive ionizable tertiary amine group (PI') is located in close proximity to negatively charged residues; Glu172 and Asp126 (Figure 3D).

**Figure 4:**
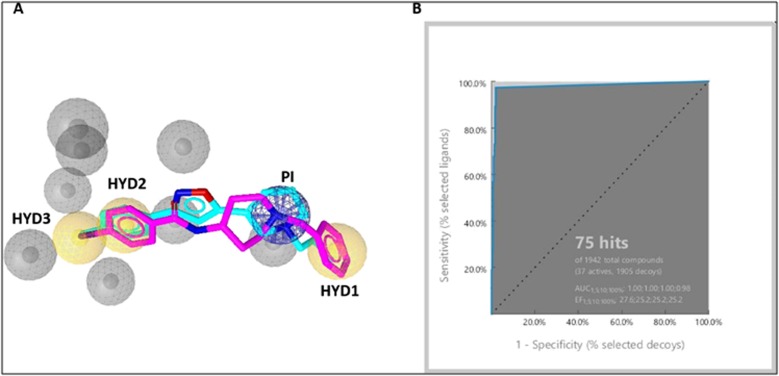
(A) Model of shared features pharmacophore of 5HK1 and 5HK2 protein structure ligands. Mapping ligands, PD144418 (cyan) and 4-IBP (pink) on the final pharmacophore model is shown. The pharmacophore features were represented in LigandScout by color codes in which, hydrophobic, ionizable positive charge and exclusion volume are depicted as yellow spheres, blue stars and gray spheres, respectively. HYD and PI stand for hydrophobic and positive ionizable features, respectively. (B) The receiver operating characteristics (ROC) validation curve of pharmacophore model.

The alignment of these two pharmacophores to extract the shared features resulted in the final pharmacophore containing four key features three hydrophobic regions, a positive ionizable group beside eight exclusion volume spheres as shown in Figure 4A. All the pharmacophore features are around targetable active site of σ1 receptor with well-defined geometrical distances that are essential for the binding to the receptor. Interestingly, this pharmacophore model with a nitrogen atom centring two hydrophobic sides is in agreement with the typical pharmacophore features for σ1 receptor binders [64]. Therefore, compounds mapping on these features might have potential to bind to σ1 receptors with high affinity as well as to modulate their function.

In order to verify the derived structure-based pharmacophore model, virtual screens were performed on two datasets of small molecules of actives and decoys. The main reason for using this method was to validate the ability of the pharmacophore to predict the active molecules from inactive molecules [65]. The active set was composed of 37 compounds collected from the literature and the decoys were 1905 compounds with unknown activity toward σ1 receptor. After screening, the receiver operating characteristics (ROC) graphs were generated, and the area under the curve (AUC) as well as the enrichment factor (EF) was calculated. The early enrichment factor (EF1%) was 27.6 with an ideal ROC-AUC value of 1 indicating that our pharmacophore model was rational for virtual screening as it was able to predict 36 active compounds from the total of 37 active compounds (Figure 4B).

In our study, a GPCR-focused chemical library of 8124 molecules was screened against the derived pharmacophore model. Among those, 159 compounds were found to fit the pharmacophore features. The candidate molecules were ranked according to the pharmacophore-fit score which reflected how the molecules fit the features of the pharmacophore queries used for the virtual screening. The pharmacophore-fit scores for these compounds ranged from43.41 to 40.77.Subsequently, the identified candidate compounds were subjected unbiasedly to docking-based virtual screening to filter them further based on the binding energy score. To filter the obtained 159 compounds, these molecules were subjected to docking based high-throughput screening against the active site of human σ1 receptor structure using Autodock Vina in PyRx programs. The screening resulted in the identification of 45 candidate compounds with low binding affinity scores (ranged from -12.4 to -10.1 Kcal/mole) in comparison to the co-crystal ligand (-10.0 Kcal/mole) that was added to dataset.

Analyses of the ADMET (absorption, distribution, metabolism, elimination and toxicity) properties are a crucial step in drug design. Lipinski's rule of five in Swiss ADME web server was applied to filter the 45 compounds and select the potential hits [59]. The Swiss ADME web server restrictions were as follows; molecular weight ≤ 500, MlogP≤4.5, topological polar surface area (TPSA) ≤ 5, number of rotatable bonds ≤ 5, hydrogen bond acceptors should be less than 10, hydrogen bond donors should be less than 5. Since the site of action of σ1 receptor ligands is mostly in the brain, the candidate compounds must be able to penetrate the blood brain barrier (BBB). The level of aqueous solubility was also considered as it is necessary to facilitate the in vitro and in vivo characterization of potential hits. In addition, the hepatotoxicity and mutagenicity profiles were also analyzed simultaneously for the 45 compounds using ProTox-II web server [60]. Compounds only with the following properties were considered as candidate compounds. The compound must show zero violation of Lipinski's rule of five; good aqueous solubility, an ability to penetrate the BBB, be devoid of mutagenicity, and unlikely to cause dosedependent hepatotoxicity. After applying these filters, only five compounds with diverse chemical scaffolds were picket out. The chemical structure, pharmacophore-fit score and binding free energy score of these compounds are listed inTable 1.

**Figure 5:**
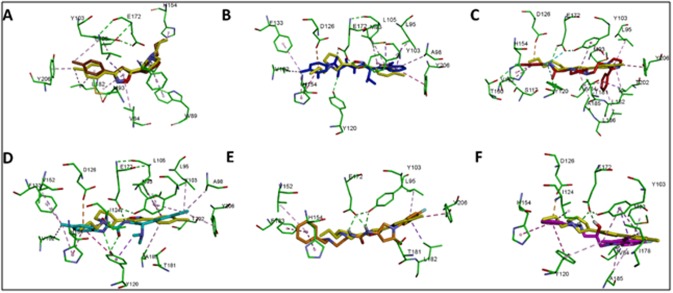
Binding modes of candidate compounds to human σ1 receptors. (A) Best docked conformation of PD144418 (brown) overlapped with co-crystal ligand (yellow). (B) Best docked conformation of F5478-0036(blue) overlapped with co-crystal ligand (yellow). (C)Best docked conformation of F6368-0290 (red) overlapped with co-crystal ligand (yellow). (D) Best docked conformation of F2291-0434 (cyan) overlapped with co-crystal ligand (yellow). (E) Best docked conformation of F2024-1993 (orange) overlapped with co-crystal ligand (yellow). (F)Best docked conformation of F3352-0087 (pink) overlapped with co-crystal ligand (yellow).

In order to further refine the retrieved hits, candidate compounds were docked into to the active site of human σ1 receptor using Autodock Vina 1.1.2 program. The active binding site was defined based on the bound ligand, PD144418 in an X-ray structure of the σ1 receptor (PDB: 5HK1).Before performing the molecular docking, we first validated our docking approach by extracting the co-crystal ligands, PD144418, from the σ1 receptor structure and then redocked it into the active binding site to verify the ability of the docking program and protocol to reproduce the bioactive conformation of PD144418. The resulted docking pose from this exercise with the lowest binding free energy score adapted the same binding mode as the co-crystal ligand Figure 5A. These results illustrate the robustness of the docking program and protocol.

**Figure 6:**
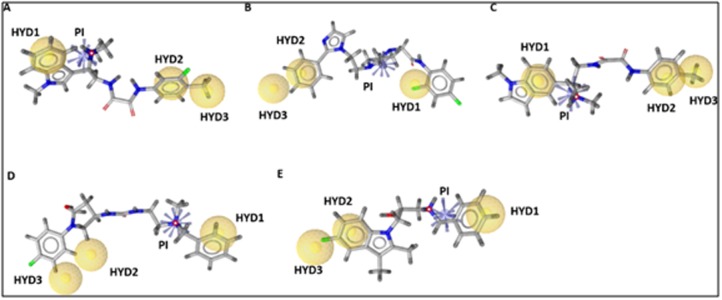
Fit of the (A) F5478-0036, (B) F6368-0290, (C) F2291-0434, (D) F2024-1993 and (E) F3352-0087 to the structure-based pharmacophore model. The candidate compounds fit all the four features and all of the excluded volumes. The pharmacophore features were represented in LigandScout by color codes in which, hydrophobic, ionizable positive charge and exclusion volume are depicted as yellow spheres, blue stars and gray spheres, respectively. HYD and PI stand for hydrophobic and positive ionizable features, respectively.

The docking scores of the five candidate compounds (-11.5-10.2 Kcal/mole) were lower than that of co-crystal ligand (-10.00 Kcal/mole) as shown in Table 1. The best binding poses for the candidate compounds are shown in Figure 5B-F. These compounds adapted the same binding modes as the reported co-crystal ligands within the active site of σ1 receptor. The candidate compounds form extensive hydrophobic interactions with hydrophobic residues within the active site. Importantly, the aromatic rings of every candidate compound forms a π-π stacking interaction with Tyr103 amino acid residue. This interaction is shown to be essential for the binding to σ1 receptor [14] Moreover, the charge-charge interaction between Tyr103 and Glu172 is also observed in our docking results with all compounds which is suggested to be necessary to stabilize the orientation ofTyr103in binding site as well as the ligands binding [14]. The pharmacophore mapping of five candidate compounds on the derived pharmacophore model is depicted in Figure 6. The superimposing of these candidate compounds on the pharmacophore model indicates that the candidate compounds can produce perfect mapping with pharmacophore model. The compounds have mapped all pharmacophore features including the exclusion volume and scored good fit values.

## Conclusion

In this present work, we attempted to identify new σ1 receptor ligands via a combined pharmacophore and docking-based virtual screening with drug-likeness and ADMET analysis. The structurebased pharmacophore model was established using two co-crystal complexes of σ1 receptor bound to agonist and antagonist ligands. The model was composed of three hydrophobic features and a positive ionizable area and was used as a 3D query to screen a focused GPCRs chemical library. Prior to screening of chemical library, the model was validated using active and decoy sets method to evaluate its eminence to identify reliable compounds. Five candidate compounds possessing different chemical scaffolds with excellent in silico binding scores, drug-likeness, hepatotoxicity and mutagenicity profiles were identified. In summary, our study suggested that these five candidate compounds may act as potential σ1 receptor ligands with high binding affinity.

## Figures and Tables

**Table 1:** Names, Chemical structures, pharmacophore-fit scores and binding energy of the candidate compounds

Compound	Pharmacophore Fit Score	Binding Energy (Kcal/mole)
F5478-0036	42.22	-11.5
F6368-0290	42.05	-11.5
F2291-0434	42.7	-11.3
F2024-1993	42.79	-10.8
F3352-0087	41.77	-10.2
